# Spontaneous regression of breast lymphoproliferative disorders after withdrawal of methotrexate in rheumatoid arthritis patients with Epstein–Barr virus infection: a case report and review of the literature

**DOI:** 10.1186/s13256-022-03274-1

**Published:** 2022-02-07

**Authors:** Ayumi Ogawa, Tsuyoshi Nakagawa, Yuichi Kumaki, Tokuko Hosoya, Goshi Oda, Mio Mori, Tomoyuki Fujioka, Kazunori Kubota, Iichiro Onishi, Hiroyuki Uetake

**Affiliations:** 1 Department of Breast Surgery, Tokyo Medical and Dental University, 1-5-45 Yushima, Bunkyo-ku, Tokyo, 113-8510 Japan; 2 Departments of Diagnostic Radiology, Tokyo Medical and Dental University, Tokyo, Japan; 3 Comprehensive Pathology, Tokyo Medical and Dental University, Tokyo, Japan; 4 Department of Surgical Specialties, Tokyo Medical and Dental University, Tokyo, Japan

**Keywords:** Methotrexate-associated lymphoproliferative disorders (MTX-LPD), Other iatrogenic immunodeficiency-associated lymphoproliferative disorders (OIIA-LPD), Breast lymphoma, Epstein–Barr virus (EBV)

## Abstract

**Background:**

Lymphoproliferative disorder (LPD) has been shown to occur after treatment with methotrexate (MTX). Currently, MTX-LPD has become widely recognized, but its mechanism and prognostic factors remain unclear.

**Case presentation:**

We report the first case of Epstein–Barr virus (EBV)-associated MTX-LPD of the breast. A 63-year-old Asian woman with long-term rheumatoid arthritis presented to our facility with intermittent fever. A physical examination revealed a 3-cm lump in her left breast. She had been taking MTX for the past 15 years. Laboratory studies revealed slightly elevated levels of EBV-viral capsid antigen antibody immunoglobulin G and EBV nuclear antibody. Contrast-enhanced computer tomography revealed a mass in the left breast, a subcutaneous nodule in the abdomen, a mass in the left lung, and a nodule in the left retroperitoneum. The definitive diagnosis was consistent with MTX-LPD merging into an EBV-positive, diffuse large B-cell lymphoma. Six months following the withdrawal of MTX, the breast mass had markedly shrunk and the patient remained in good health for 1 year with no evidence of relapse of LPD.

**Conclusion:**

MTX-LPD rarely occurs in the breast, and it is difficult to diagnose because there have only been six reported cases of breast MTX-LPD reported in the literature. EBV-positive MTX-LPD tends to regress spontaneously after MTX withdrawal, and our case also had similar results. It is important to make an appropriate diagnosis of MTX-LPD of the breast based on imaging and pathology to determine the appropriate treatment protocol for this rare disorder.

## Background

Lymphoproliferative disorder (LPD) has been shown to occur in some patients who are immunosuppressed due to treatment with methotrexate (MTX), hence the name MTX-associated lymphoproliferative disorder (MTX-LPD). It is known that rheumatoid arthritis (RA) patients have a high risk of developing malignant lymphoma, but epidemiological evidence of an association between the use of MTX and development of lymphoma is still unclear [1]. At present, LPD that arises in patients treated with immunosuppressive drugs, including biologic drugs for autoimmune diseases, is categorized as “other iatrogenic immunodeficiency-associated lymphoproliferative disorder (OIIA-LPD)”, other than in post-transplant lymphoproliferative disorder, according to the World Health Organization (WHO) Classification of Tumors of Hematopoietic and Lymphoid Tissues, 4th edition (2008).

Unlike usual malignant lymphomas, MTX-LPDs are relatively common in extranodal sites such as the skin, lungs, and oral and pharyngeal cavities; genetic factors and accumulated inflammation are characteristic of MTX-LPD [1]. Moreover, the relationship between Epstein–Barr virus (EBV) infection and MTX-LPD has been previously indicated by the WHO classification [2]. In some cases, MTX-LPD has shown partial regression in response to drug withdrawal alone, and the majority of this response has occurred in EBV-positive cases [3]. Currently, MTX-LPD has become widely recognized, and MTX is more often discontinued when MTX-LPD is suspected. However, MTX-LPDs of the breast are very rare and difficult to diagnose, because only six cases have been reported thus far [4–9]. To the best of our knowledge, this is the first case report on EBV-positive LPD in the mammary gland, which disappeared spontaneously after MTX was discontinued.

## Case presentation

A 63-year-old Asian woman presented with intermittent fever of approximately 38.0 °C, which had lasted for 2 weeks and did respond to antipyretics. She had no night sweating or weight loss. She had been suffering from RA for 37 years and had been taking MTX for more than 15 years. The latest medication doses were 14 mg/week of MTX, 5 mg/day of prednisolone, and 1 mg/day of tacrolimus. She did not receive any other immunosuppressive medication. Other medical history included diabetes, atypical mycobacteriosis, and hepatitis B and there was no family history of lymphoproliferative disorders or malignancies. The patient did not smoke or drink alcohol.

**Fig. 1:**
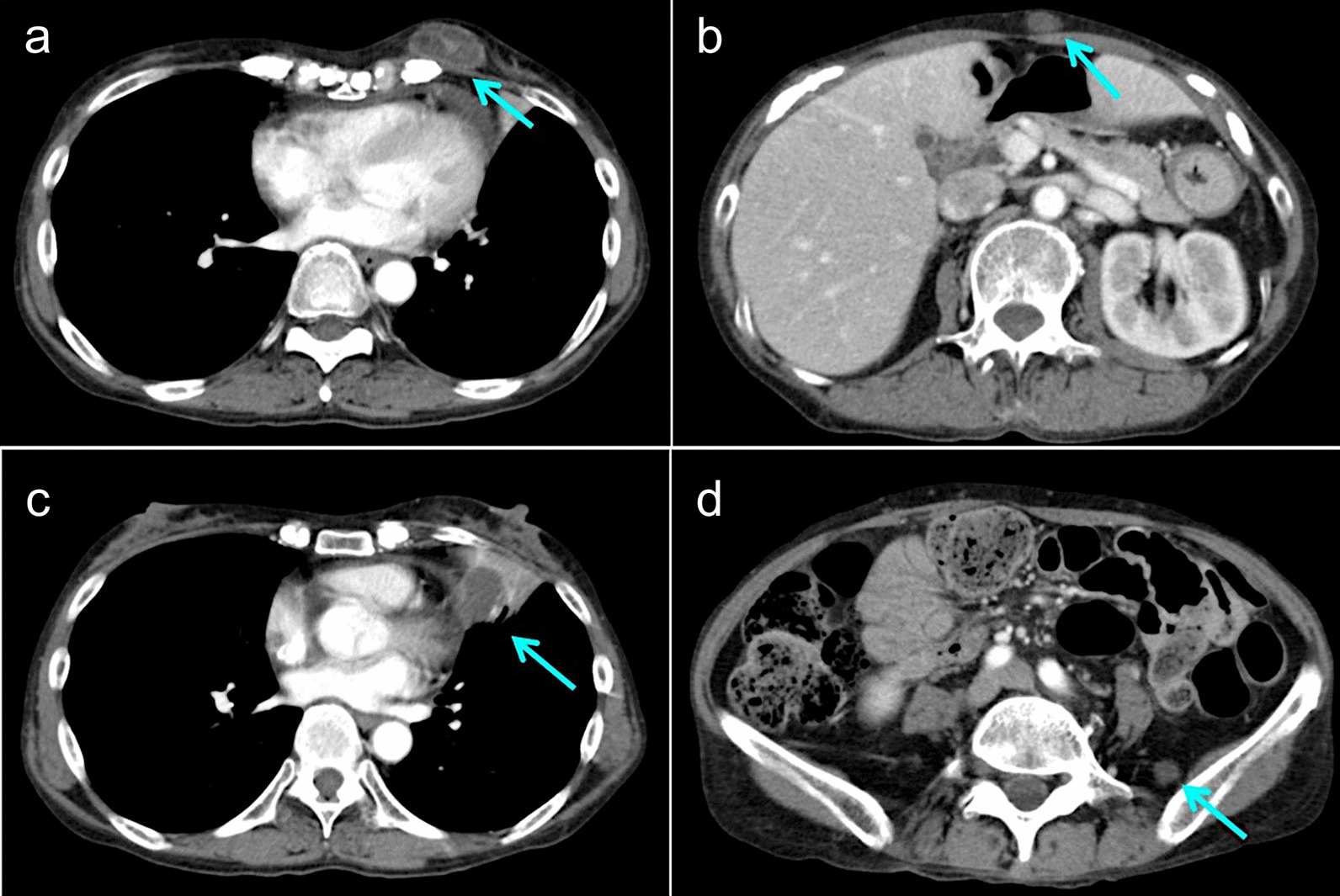
Contrast-enhanced computed tomography scan imaging **a** a mass in the breast (arrow), **b** a subcutaneous nodule in the abdomen (arrow), **c** a mass in the left lung (arrow), **d** a nodule in the left retroperitoneum (arrow)

At the time of admission, she had fever of 38.0 °C. Physical examination showed a 3-cm palpable mass in the left breast. There were no visible skin changes and no palpable lymph nodes. Laboratory studies (Table 1) revealed slightly elevated levels of C-reactive protein and soluble interleukin-2 receptor (sIL-2R). EBV-viral capsid antigen antibody immunoglobulin G and EBV nuclear antibody (EBNA) were also increased. We performed contrast-enhanced computer tomography of her chest and abdomen for general evaluation. Results revealed a mass in the left breast, a subcutaneous nodule in the abdomen, a mass in the left lung, and a nodule in the left retroperitoneum (Fig. 1). We suspected that these tumors were associated with administration of MTX; thus, MTX was discontinued immediately, and the patient was hospitalized for medical examination.

**Table 1:** Summary of the laboratory data of the patient on admission

Investigations	Values	Reference range	Units
White blood cells	8.3	3.3–8.6	× 1000/μL
Neutrophils	67.0	41.7–74.1	%
Hemoglobin	13.9	11.6–14.8	g/dL
Platelet counts	32.7	15.8–34.8	× 10000/μL
PT-INR	1.1	0.9–1.1	
APTT	26.2	24.5–39.7	seconds
Total protein	7.4	6.6–8.1	g/dL
Albumin	4.0	4.1–5.1	g/dL
Blood-urea-nitrogen	29	8–20	mg/dL
Creatinine	0.78	0.46–0.79	mg/dL
Na	142	138–145	mEq/L
K	4.7	3.6–4.8	mEq/L
Lactate dehydrogenase	289	124–222	U/L
Aspartate aminotransferase	22	13–30	U/L
Alanine aminotransferase	13	7–23	U/L
Total bilirubin	0.7	0.4–1.5	mg/dL
C-reactive protein	1.47	< 0.14	mg/dL
HbA1c	6.6	4.9–6.0	%
sIL-2R	620	145–519	U/mL
EBV-VCA IgG	160	< 10	Fold
EBV-VCA IgM	< 10	< 10	Fold
EBV-EBNA	40	< 10	Fold

*APTT* activated partial thromboplastin time, *EBNA* EBV-nuclear antigen, *EBV* Epstein–Barr virus, *EBV-VCA* Epstein–Barr virus-viral capsid antigen, *HbA1c* glycated hemoglobin, *IgA* immunoglobulin A, *IgG* immunoglobulin G, *PT-INR* prothrombin time-international normalized ratio, *sIL-2R* soluble interleukin-2 receptor

**Fig. 2:**
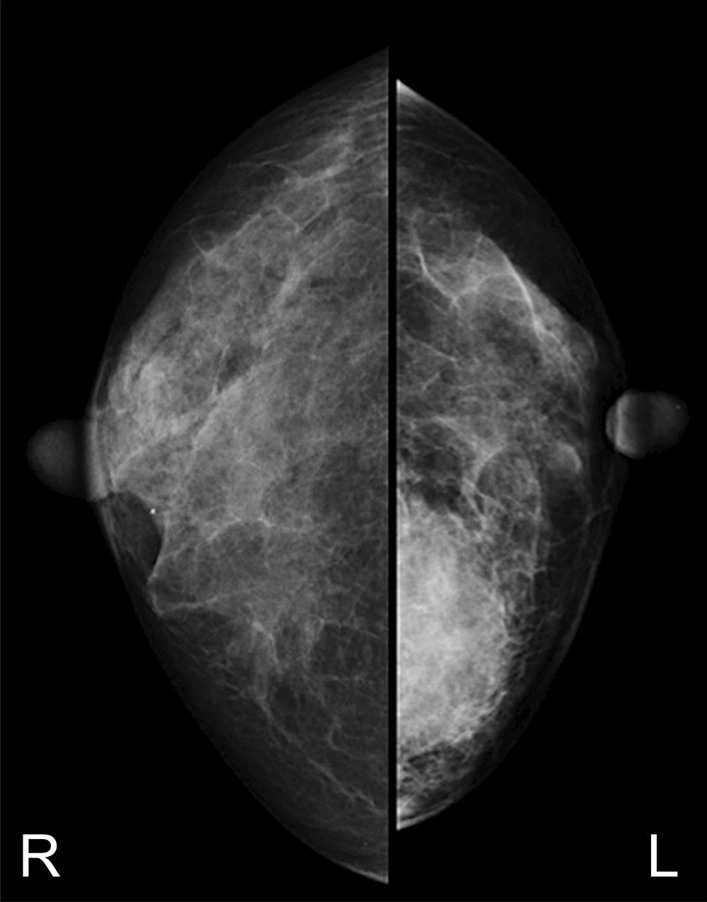
Craniocaudal mammography imaging. A high-density mass with indistinct margins in the internal region of the left breast

**Fig. 3:**
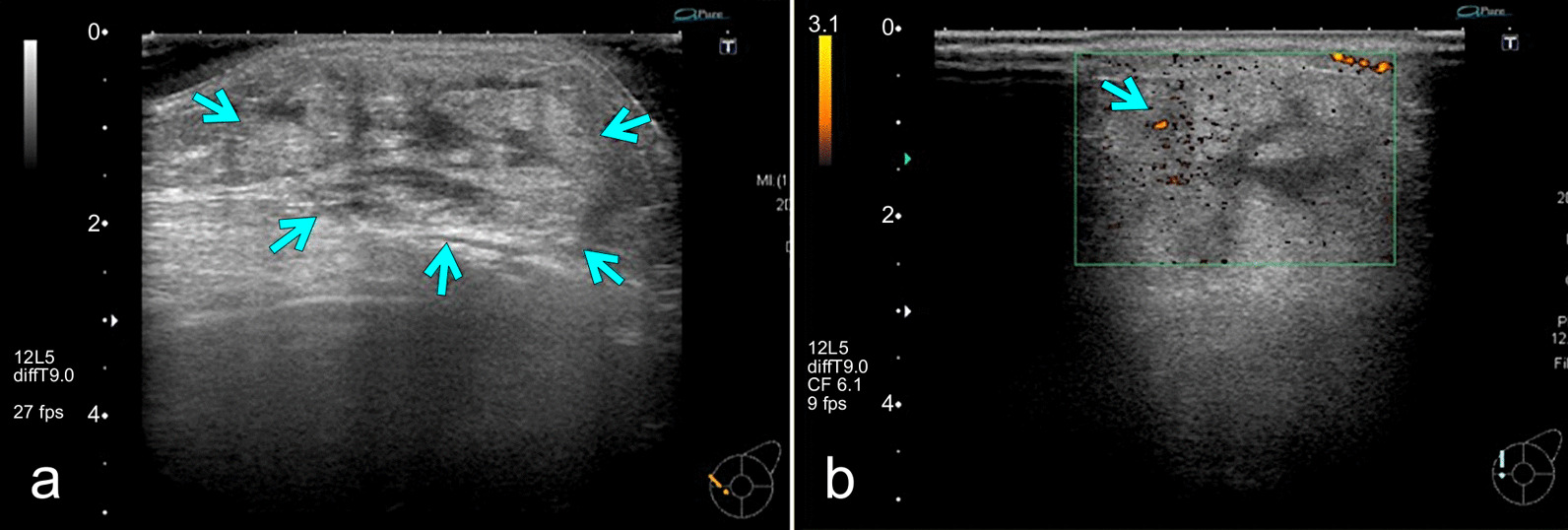
Ultrasonography (US) of the left breast. One week after withdrawal of methotrexate, **a** US showed a 4.5-cm heterogeneous hyperechoic mass in the internal region of the left breast (arrow). **b** The mass had a low echo area spread like a cord, and blood flow was partially observed along the low echo area (arrow)

**Fig. 4.**
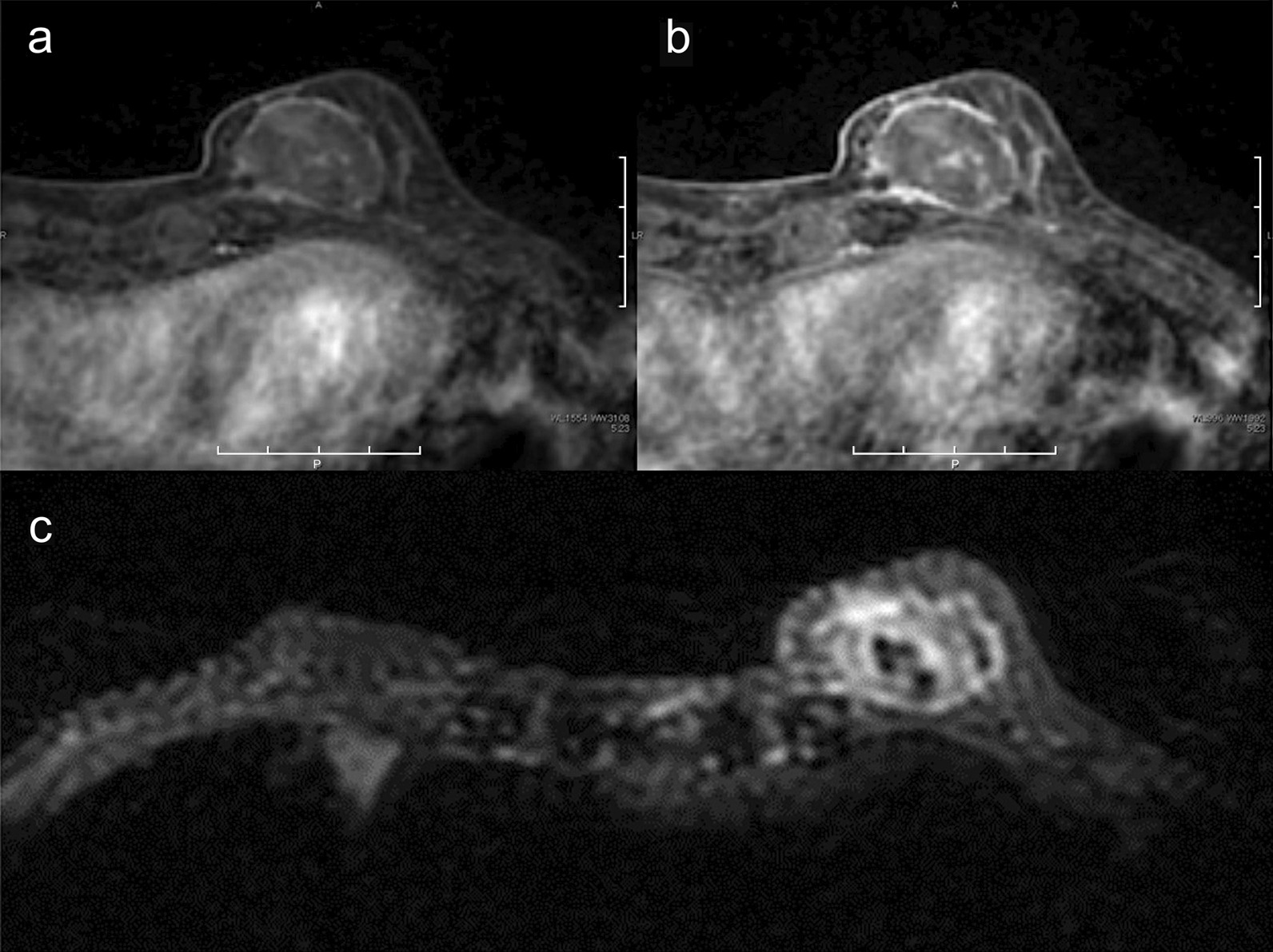
Contrast-enhanced breast magnetic resonance imaging (MRI). **a** Initial phase of dynamic contrast-enhanced MRI, **b** Delayed phase of dynamic contrast-enhanced MRI; the contrast effect appeared only on margins and part of the interior, **c** Fat-suppressed T2 weighted image; the mass showed high signal

One week after MTX withdrawal, imaging evaluation was performed. Craniocaudal and mediolateral oblique mammography showed a high-density mass with undefined margins in the internal lower left breast quadrant (Fig. 2). Ultrasonography (US) showed a 4.5-cm heterogeneous hyperechoic mass in the internal region of the left breast, which had a low echo area spread like a cord, and blood flow was partially observed along the low echo area (Fig. 3). Contrast-enhanced bilateral breast magnetic resonance imaging (MRI) showed a well-defined mass in the same area. The contrast effect appeared only on margins and part of the interior, and the non-imaged mass showed a high signal in fat suppressed T2 weighted image (Fig. 4).

**Fig. 5:**
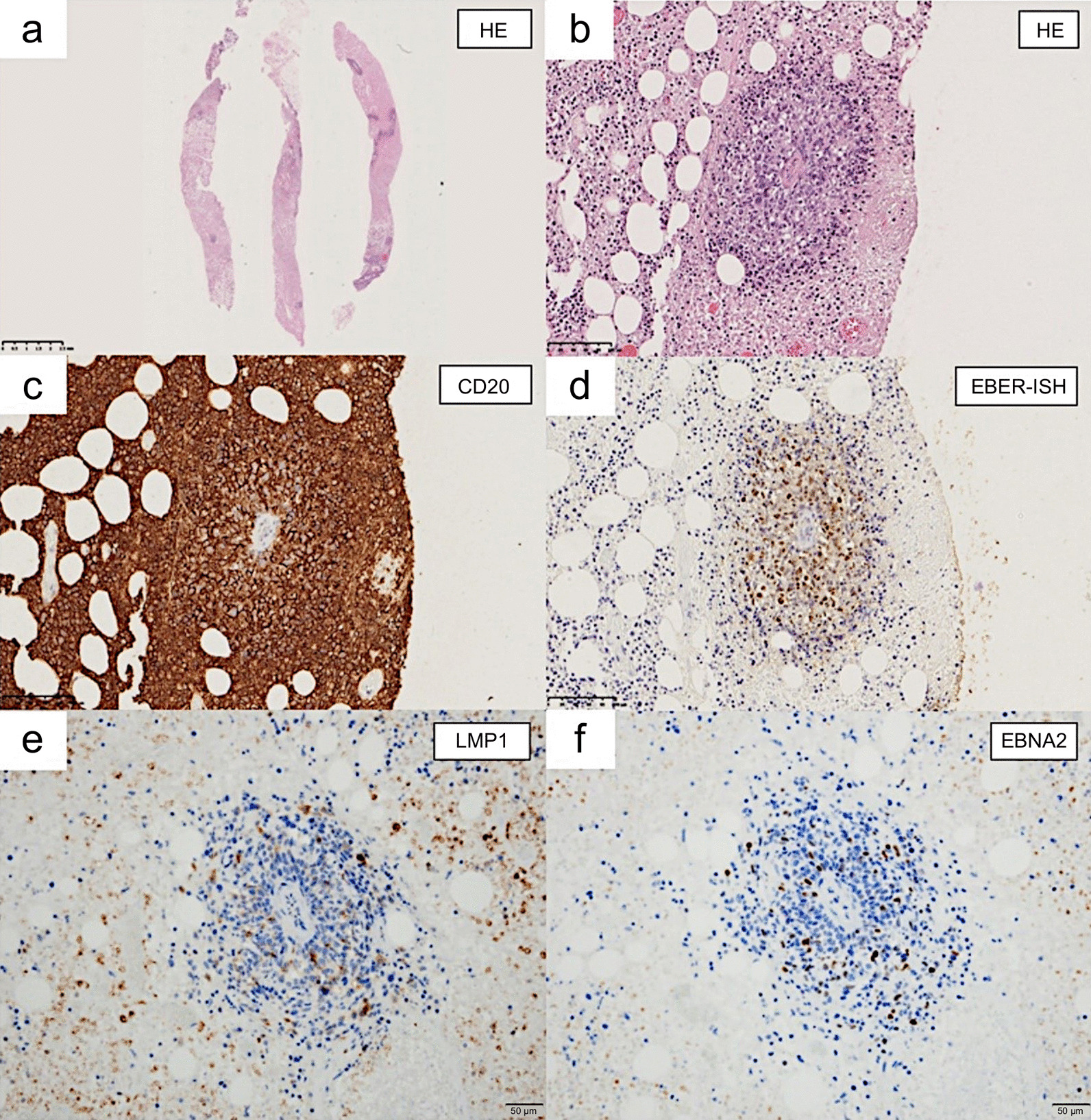
Pathological results from ultrasound-guided core needle biopsy of the left breast mass. Biopsies were mainly composed of necrotic tissue, and atypical lymphoid cells were around the blood vessel (**a** and **b** HE). Lymphoid cells were positive for CD20 (**c**), EBER-ISH (**d**), LMP1 (**e**), and EBNA (**f**). Magnification: **a** × 20; **b**–**d** × 200; **e**, **f** × 400. *EBER-ISH* Epstein–Barr virus infection detected by *in situ* hybridization, *EBNA* Epstein–Barr virus nuclear antibody, *HE* hematoxylin & eosin, *LMP1* latent membrane protein 1

Subsequently, an ultrasound-guided core needle biopsy was performed on the left breast mass. Biopsies from the breast mass were primarily composed of necrotic tissue and revealed the proliferation of atypical lymphocytes with enlarged nuclei around the blood vessel. On immunohistochemical staining, lymphoid cells were positive for CD20, CD79a, and BCL-2, but negative for CD3, CD7, and CD10. EBV-encoded RNA1 (EBER1), latent membrane protein 1 (LMP1), and EBV-nuclear antigen 2 (EBNA2) were positive, and the tumor was thus categorized as latency type III EBV infection (Fig. 5).

**Fig. 6:**
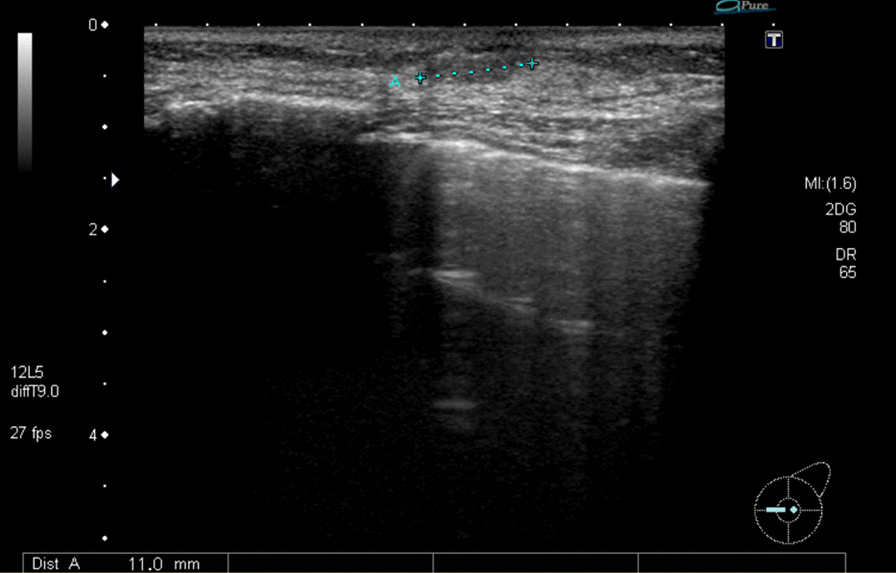
Ultrasonography (US) of the left breast. Six months after withdrawal of methotrexate, the mass had markedly shrunk like a scar

The tumors began to shrink after MTX was discontinued. Based on these clinical course and pathological findings, the definitive diagnosis was consistent with MTX-LPD merging into an EBV-positive, diffuse large B-cell lymphoma (DLBCL). One month following withdrawal of MTX, the breast mass had become smaller, about 2-cm in diameter on US. Six months later, the mass had markedly shrunk like a scar (Fig. 6), and the patient remained in good health for 1 year after the withdrawal of MTX with no evidence of relapse of LPD. To treat the patient’s RA, iguratimod, salazosulfapyridine, and bucillamine were prescribed instead of MTX, and they provided adequate RA control. The patient is currently on a follow-up plan involving core laboratory studies, including sIL-2R every 3 months, and breast US every 6 months to 1 year.

## Discussion and conclusions

MTX has generally become the first-line drug for the treatment of RA since its adoption in the 1980s. It provides benefits for a considerable number of RA patients but is unfortunately associated with several adverse side effects [10]. The first association between MTX and lymphoma was reported in 1991 when Ellman *et al.* uncovered lymphoma developing in an RA patient who was administered MTX [11]. After similar cases had been reported, this condition became known as MTX-LPD.

In rare cases, MTX-LPD occurs in the breast, and we reviewed only seven such cases, including the present case [4–9] (Table 2). All patients had a history of RA treated with MTX, and two patients received other anti-rheumatoid agents in combination with MTX. All patients were female. One case occurred in the nipple-ring complex, and another case occurred independently in the mammary gland. At the time of lymphoma diagnosis, the median age was 69 years (range, 63–79 years). The majority of patients had long-standing RA (median duration 20 years; range, 14–37 years). The periods of MTX administration varied from 7 months to 17 years. Six patients had DLBCL, and one patient had small B-cell lymphoma (marginal zone lymphoma). Only the present case was positive for EBV infection detected by *in situ* hybridization (EBER-ISH). All patients ceased MTX treatment at the time of MTX-LPD diagnosis. Two patients had confirmed remission with only discontinuation of MTX. One case relapsed after MTX withdrawal and initiation of chemotherapy. Finally, all patients had reduced pattern or regression, and no deaths were reported.

**Table 2:** Clinical and pathological findings in 7 cases of MTX-LPD occurring in the breast in RA

No.	Age/Sex	History of RA	Duration of MTX	Other RA treatment	Mass location	Pathology	EBV	Therapy and response	Follow up period	References
1	69/F	24 years	17 years	Etanercept (7 years)	Breast Lung Retroperitoneum	DLBCL	(−)	**W** + **C**→PR	3.5 months	Pattanaik *et al.* [4]
2	76/F	–	7 months	–	Breast	DLBCL	(−)	**W** + **C**	–	Hashimoto *et al.* [5]
3	63/F	20 years	10 years	–	Breast Subcutis	DLBCL	–	**W**→CR	1 year	Fujimitsu *et al.* [6]
4	79/F	14 years	2 years	Infliximab (2 years)	Bilateral breast Lymph nodes Subcutis	small B-cell lymphoma (marginal zone lymphoma)	–	**W** + **C**→CR	–	Costa *et al.* [7]
5	67/F	–	–	–	NAC of the breast	DLBCL	–	**W**→PD→**C**→PR	3 months	Matsubayashi *et al.* [8]
6	77/F	17 years	13 years	–	Breast Lymph nodes	DLBCL	(−)	**W**→SD→**C**→CR	16 months	Miyake *et al.* [9]
7	63/F	37 years	More than 15 years	–	Breast Subcutis Lung Retroperitoneum	DLBCL	(+)	**W**→CR	1 year	Current case report

*C* chemotherapy, *CR* complete remission, *DLBCL* diffuse large B-cell lymphoma, *EBV* Epstein–Barr virus, *MTX* methotrexate, *NAC* nipple-areolar complex, *PD* progressive disease, *PR* partial remission, *RA* rheumatoid arthritis, *SD* stable disease, *W* withdrawal of M

To our knowledge, this is the first case of EBV-positive MTX-LPD occurring in the breast. The relationship between EBV infection and MTX-LPD has been previously indicated, and it is conceivable that EBV is reactivated in the lesions [2]. In addition, 30–50% of MTX-LPD cases in RA patients have EBV positivity [12]. It has been suggested that this is due to the immunosuppressive effects of MTX, which leads to the reduced function of cytotoxic T cells, thereby resulting in the latent proliferation of EBV-infected B cells [13]. Sometimes, MTX-LPD has shown partial regression in response to drug withdrawal alone, and the majority of this response has occurred in EBV-positive cases [3].

According to the immunohistochemical expression patterns of EBV latent RNA and proteins, EBV-associated malignancies are categorized into three groups [14]: latency type I (EBER +, LMP1 −, and EBNA2 −), including Burkitt lymphoma; latency type II (EBER +, LMP1 +, and EBNA2 −), including Hodgkin lymphoma and nasopharyngeal carcinoma; and latency type III (EBER +, LMP1 +, and EBNA2 +), including LPD arising in immunocompromised patients. However, the relationship between MTX-LPD and the types of EBV latency is unclear, as only a few cases of EBV latency in MTX-LPD have been reported. The present case showed EBER +, LMP1 +, and EBNA2 + and was categorized as latency type III. Miyazaki *et al.* reported that non-Hodgkin’s lymphoma type MTX-LPD, especially in the latency type III group, showed regression, and that analysis of EBV latency is useful for deciding an optimal therapeutic strategy [15]. This patient with latency type III tumor also had similar regression results. On the contrary, Ishida *et al.* interestingly reported EBV-positive MTX-LPD diagnosed in a swollen tonsil biopsy [16]. It spontaneously regressed after withdrawal of MTX, and the follow-up specimens from the tonsil became EBV-negative and free of LPD. LPD regression led to negative EBER-ISH, suggesting the possibility that previously negative cases may have once been positive, depending on the time of diagnosis. Thus, it may be difficult to consider prognosis with EBER-ISH of the tissue. Currently, peripheral blood EBV-DNA is being used for examination [17]. Au *et al.* reported that plasma-derived EBV-DNA is valuable both as a tumor marker and prognostic biomarker in EBV-positive lymphomas [18]. Unfortunately, EBV-DNA was not measured in this case, but it will be used for further studies in the future.

As breast tissue is on the body surface, it can be easily evaluated by US, and US is relatively common for observing breast tissue. Primary breast lymphoma usually displays a very low echo with a well-demarcated mass, which reflects its high cell density [19]. In contrast, this case revealed mainly hyperechoic mass, and the tumor had a low echo area spread like a cord and blood flow along the low echo area, 1 week after withdrawal of MTX. In the pathology specimens of the breast, which were performed at the same time, there were mostly necrotic areas and partially viable lymphoid cells around the blood vessels. Because the low echo area indicates uneven cell density, in contrast with the ultrasound and pathological findings, the majority of the high echo area reflects the wide necrotic area, and the cord-like low echo area with blood flow reflects the blood vessels and lymphoid cells. This is supported by the MRI findings, which showed a contrast effect only on the margins and part of the interior of the non-imaged mass. Moreover, the non-imaged mass revealed high signal in fat-suppressed T2 weighted image, which indicates that the inside of the tumor is necrotic. On the other hand, the previous six patients with MTX-LPD of the breast had heterogeneous hypoechoic masses. Unfortunately, in this case, the image could not be evaluated before MTX discontinuation, and the previous six reports did not describe the temporal context between image evaluation and MTX discontinuation in detail. However, presuming that the withdrawal of MTX leads to tumor necrosis and changes a hypoechoic mass into a hyperechoic mass, image evaluation before and after MTX discontinuation may serve as a predictor and determinant of therapeutic efficacy.

Finally, here are two interesting reports from Japan. Shimizu *et al.* reported that two cases of MTX-LPD of the lung with an EBER-negative pattern showed improvement before discontinuation of MTX [20]. Both lung diagnostic specimens were mainly composed of “necrosis.” Ejima-Yamada *et al.* also reported that EBV infection is associated with the hypermethylation of apoptosis-related genes, which leads to tumor regression after the withdrawal of MTX [1, 21]. The disease progression, resolution, or recurrence may occur regardless of MTX continuation or discontinuation. Prognostic factors remain unclear, and it is not certain how EBV and necrosis are involved. However, we believe that imaging and pathological findings of necrosis early in the course of the disease are among the prognostic factors to consider. In particular, when MTX-LPD is suspected, it is crucial to screen for EBV infection and repeat image evaluation in order to avoid undetected necrosis.

We have reported the first case of EBV-positive MTX-LPD of the breast. MTX-LPD rarely occurs in the breast and is difficult to diagnose. EBV-positive MTX-LPD tends to regress spontaneously after withdrawal of MTX, and our case also had similar results. It is important to make an appropriate diagnosis based on images and pathology in order to determine appropriate treatment protocol.

## Data Availability

Not applicable.

## Abbreviations

LPD: Lymphoproliferative disorder
MTX: Methotrexate
EBV: Epstein–Barr virus
OIIA-LPD: Other iatrogenic immunodeficiency-associated lymphoproliferative disorders
RA: Rheumatoid arthritis
WHO: World Health Organization
sIL-2R: Soluble interleukin-2 receptor
EBNA: EBV nuclear antibody
US: Ultrasonography
MRI: Magnetic resonance imaging
EBER1: EBV-encoded RNA1
EBNA2: EBV-nuclear antigen 2
DLBCL: Diffuse large B-cell lymphoma
